# MMP9 and IGFBP1 Regulate Tumor Immune and Drive Tumor Progression in Clear Cell Renal Cell Carcinoma

**DOI:** 10.7150/jca.48664

**Published:** 2021-02-22

**Authors:** Tianbo Xu, Su Gao, Jingchong Liu, Yu Huang, Ke Chen, Xiaoping Zhang

**Affiliations:** 1 Department of Urology, Union Hospital, Tongji Medical College, Huazhong University of Science and Technology, 1277 JieFang Avenue, Wuhan 430022, China.; 2 Department of Geriatrics, Union Hospital, Tongji Medical College, Huazhong University of Science and Technology, 1277 JieFang Avenue, Wuhan 430022, China.; 3 Institute of Gerontology, Union Hospital, Tongji Medical College, Huazhong University of Science and Technology, 1277 JieFang Avenue, Wuhan 430022, China.

**Keywords:** clear cell renal cell carcinoma, MMP9, IGFBP1, biomarker, tumor-infiltrating immune cells

## Abstract

Immunotherapy is a novel approach and has been used in various diseases, especially in cancers. Recently, immunotherapy has gradually been used to treat advanced clear cell renal cell carcinoma (ccRCC) or metastatic ccRCC. However, the efficacy of immunotherapy is not satisfying due to the influence of the tumor microenvironment. In this study, we mainly focused on the abundance and function of tumor-infiltrating immune cells (TIICs). Monocyte and TNM stage were identified as independent prognostic factors via CIBERSORT and Cox regression analysis. Then, ccRCC patients were divided into high risk/TNM^high^Monocytes^low^ cluster and low risk/TNM^low^Monocytes^high^ cluster. Further differential gene analysis, protein-protein interaction (PPI) network, and survival analysis screened nine hub genes between the above two clusters. MMP9 and IGFBP1 were selected for further study through sample validation. Moreover, gene set enrichment analysis revealed that MMP9 and IGFBP1 were involved in tumor immune via mediating cell surface receptor signal pathway, cytokine production pathway, or monocyte signal pathway. In conclusion, these findings suggested that monocyte acted as a protective factor and MMP9/IGFBP1 played a vital role in tumor immune, which might become potential novel biomarkers and therapeutic targets for immunotherapy in ccRCC.

## Introduction

Renal cell carcinoma (RCC) is one of the most common solid tumors and makes up about 2 - 3% of all adult malignancies 1. In 2019, approximately 73,000 new RCC patients are diagnosed and more than 14,700 patients will die due to RCC in the USA 2. RCC encompasses more than 10 different histological subtypes. Clear cell renal cell carcinoma (ccRCC) is the most common subtype of RCC, which accounts for 70 - 80% of RCC cases 3,4. For localized ccRCC, surgery is the most effective treatment and can significantly prolong the survival of patients 5,6. However, there are still about 30% of newly diagnosed patients with metastasis 7,8. Besides, ccRCC is insensitive to traditional radiotherapy and chemotherapy. Molecular targeting therapy such as VEGF-tyrosine kinase inhibitors^9^ and mammalian target of rapamycin (mTOR) inhibitors 10,11 is used to treat advanced ccRCC and metastatic ccRCC patients. Unfortunately, a part of patients has not shown satisfactory improvement due to tumor recurrence and drug resistance.

Recently, immunotherapy, as a newly developed approach, has changed the treatment pattern of advanced ccRCC 12-14. Immunotherapy mainly focused on inhibiting tumor immune escape. Tumor cells can avoid being recognized and attacked by the immune system through various mechanisms including recognition of tumor specific antibodies as autoantigens, low immunogenicity of tumor cells, and tumor-induced immunosuppression, which is a vital strategy for tumor growth and progression 15,16. At present, most studies have paid attention to relieve tumor-induced immunosuppression. Tumor-induced immunosuppression is mainly regulated through two mechanisms. The first mechanism is that immunosuppressive cells accumulate around the tumor and secrete immunosuppressive factors to inhibit immune response 17-19. Another mechanism is that the tumor inhibits the activation of effector T lymphocytes via promoting the expression of immunosuppressive molecules or their receptors, such as PD-1/PD-L1 and CTLA4. PD-1/PD-L1 inhibitor is one of the most important medicines of immunotherapy. Some studies showed that PD-1/PD-L1 inhibitor could obviously benefit a subset of patients and prolong survival time in various tumors 20,21. However, it has been reported that the therapeutic effect and effectiveness of PD-1/PD-L1 antagonist was low in some tumors due to the influence and function of the tumor microenvironment (TME) 22,23. TME is composed of the vasculature, extracellular matrix (ECM), tumor cells, and a huge number of non-malignant cells 24,25. In addition, various signal molecules form a complex signal network to maintain the internal connections in the TME 26,27. Tumor-infiltrating immune cells (TIICs) are the most important part of non-malignant in the TME, which mainly include dendritic cells (DCs), natural killer (NK) cells, T cells, B cells, monocytes, macrophages, mast cells, neutrophil, and so on. Previous studies proved that TIICs played a vital role in the occurrence and development of tumors. Hu et al reveal that CD39(+)γδTregs could suppress anti-tumor immune via an adenosine-mediated pathway in colorectal cancer 28. It has been reported that neutrophils inhibited tumor growth and progression via suppressing tumor-associated inflammatory responses 29. In ccRCC, a previous report demonstrated that infiltrating CD4+ T cells promoted tumor cell proliferation via up-regulating TGF-β1 expression 30. However, the functions and molecular mechanisms of TIICs are still unclear due to the diversity of TIICs and complicacy of TME in ccRCC.

In this study, CIBERSORT, an online analytical tool for TIICs, was utilized to assess the relative abundance of 22 types of TIICs using mRNA expression data from the TCGA KIRC dataset. Then, the Cox proportional hazard regression model was constructed to divide ccRCC patients into high-risk group and low-risk group according to clinical data and abundance of TIICs. We then further screened differentially expressed genes (DEGs) between high-risk group and low-risk group and identified hub genes. Our study attempted to find key genes significantly associated with TIICs and tumor progression, which might become the potential biomarkers and therapeutic targets for immunotherapy in ccRCC.

## Material and methods

### Data collection and processing

**Figure 1:**
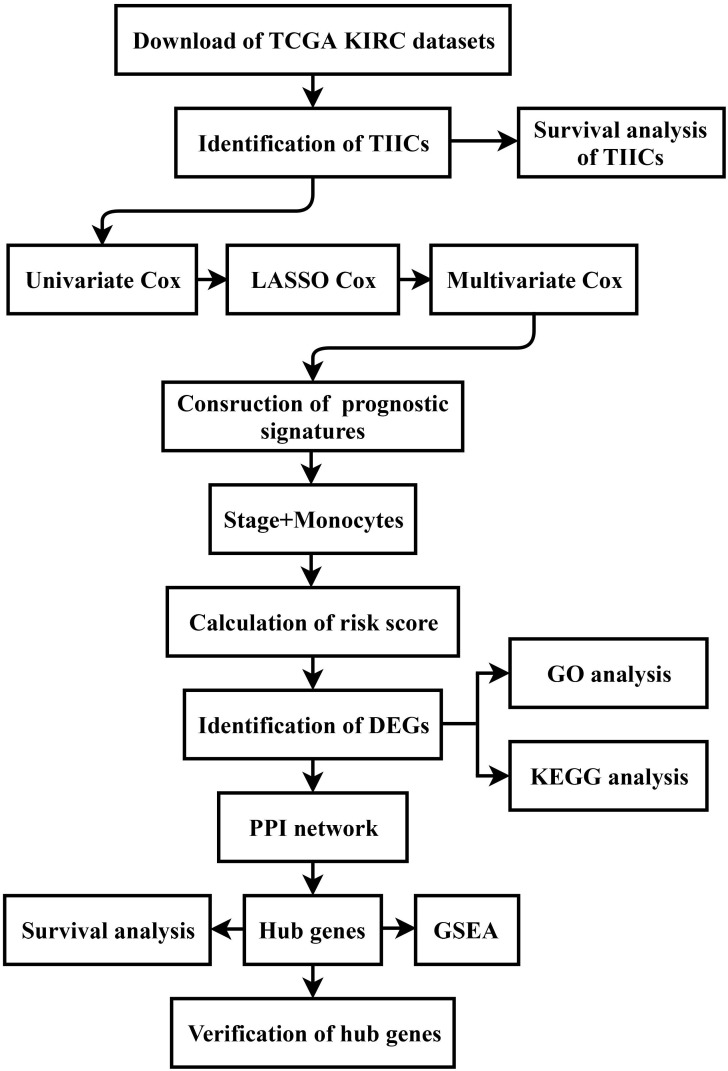
** Flow diagram of this study.** The details of data collection and analysis were exhibited in a flow diagram. TCGA: The Cancer Genome Atlas; KIRC: Kidney Renal Clear Cell Carcinoma; TIICs: tumor-infiltrating immune cells; LASSO: least absolute shrinkage and selection operator; DEGs: differentially expressed genes; GO: Gene Ontology; KEGG: Kyoto Encyclopedia of Genes and Genomes; PPI: protein-protein interaction; GSEA: gene set enrichment analysis.

All sequencing data and clinicopathological data were obtained from The Cancer Genome Atlas (TCGA) database (https://www.cancer.gov/tcga) and normalized via the R program. Moreover, the details of the data processing were described as a flow diagram (Figure 1).

### Assessment of tumor-infiltrating immune cells (TIICs)

CIBERSORT algorithm was used to infer the relative abundance of 22 TIICs in each ccRCC sample according to the normalized gene expression data. The gene expression data were submitted to the CIBERSORT web portal (http://cibersort.stanford.edu), with the algorithm run using the LM22 signature matrix at 1,000 permutations. P-value < 0.05 was considered statistically significant.

### Survival analysis of TIICs and hub genes in ccRCC

The survival data were obtained from the TCGA KIRC dataset. All ccRCC samples were divided into high group and low group according to the median abundance of TIICs or the median expression of hub genes. GraphPad Prism (version 7.0) was used to draw the survival curves of TIICs. GEPIA (http://gepia.cancer-pku.cn), an online tool, was used to perform survival analysis of hub genes. P-value < 0.05 was considered statistically significant.

### Construction of Cox proportional hazard regression model

The TCGA KIRC cohort was randomly divided into two groups (training set and test set). In the training set, univariate cox regression analysis was performed to identify TIICs, which were associated with overall survival (OS) via the “survival” package (https://CRAN.R-project.org/package=survival) in R. Then, based on the results of univariate Cox regression analysis, least absolute shrinkage and selection operator (LASSO) cox regression with a 10‐fold cross validation was used to further screen the important factors via “glmnet” 31,32 package. Next, we conducted multivariate cox regression to calculate the coefficient and construct a prognostic signature. Risk scores of ccRCC patients were calculated via the “glmnet” package based on the coefficient of each factor. We divided ccRCC patients into high-risk group and low-risk group according to the median of risk score and analyzed the prognostic role of the prognostic signature in both training set and test set.

### Identification and functional annotation of differentially expressed genes (DEGs) between high-risk cluster and low-risk cluster

The “DESeq2” 33 package was used to identify the DEGs between high-risk group and low-risk group. |log FC| > 1.0 and P-value < 0.05 were selected as cutoff criterion. Then, the “clusterProfiler” 34 package was used to perform Gene Ontology (GO) and Kyoto Encyclopedia of Genes and Genomes (KEGG) enrichment analysis in R. P-value < 0.05 was considered statistically significant.

### Construction of protein-protein (PPI) network

All DEGs were submitted to the STRING online tool (https://string-db.org) and construct a PPI network to search hub genes. Then, we utilized cytoscape software 35 to perform network analysis and calculate the degree of each DEG. Molecular Complex Detection (MCODE) plug 36 was used to screen important key modules. Genes with degree > 10 were identified as hub genes.

### Gene set enrichment analysis (GSEA)

The ccRCC samples were divided into two groups (high expression group and low expression group) based on the median expression of hub genes. The gene expression matrix was input to GSEA software 37, 38 for further enrichment analysis. Nominal P < 0.05 and false discovery rate (FDR) < 25% were selected as cutoff criteria.

### Human ccRCC samples

A total of 24 ccRCC samples were obtained from Wuhan Union Hospital between 2017 and 2018. All samples were stored at -80 °C until use. All patients signed a written consent form and the study was approved by the ethics committee of Union Hospital and Huazhong University of Science and Technology.

### RNA extraction and qRT-PCR

Total RNA was extracted from ccRCC tissue samples using Trizol Reagent (Sigma, USA). The NanoDrop Lite UV-Vis Spectrophotometer (Thermo Scientific, USA) was used to detect the concentration and purity of total RNA. Then, cDNA was synthesized using qPCR RT Kit (Vazyme, China). After that, quantitative realtime PCR (qRT-PCR) was performed to amplify cDNA with specific primers and the data were normalized to GAPDH. Primer sequences were listed as following:

GAPDH Forward: 5'-GCACCGTCAAGGCTGAGAAC-3'; GAPDH Reverse: 5'-TGGTGAAGACGCCAGTGGA-3'; MMP9 Forward: 5'-AGACCTGGGCAGATTCCAAAC-3'; MMP9 Reverse: 5'-CGGCAAGTCTTCCGAGTAGT-3'; F2 Forward: 5'-CACGGCTACGGATGTGTTCTG-3'; F2 Reverse: 5'-ACCCTCAGCACAGTTACCTTC-3'; HP Forward: 5'-CAGCACAGTCCCCGAAAAGAA-3'; HP Reverse: 5'-CAGTCGCATACCAGGTGTCC-3'; CXCL13 Forward: 5'-GCTTGAGGTGTAGATGTGTCC-3'; CXCL13 Reverse: 5'-CCCACGGGGCAAGATTTGAA-3'; IGFBP1 Forward: 5'-TTGGGACGCCATCAGTACCTA-3'; IGFBP1 Reverse: 5'-TTGGCTAAACTCTCTACGACTCT-3'; VTN Forward: 5'-CGGGGATGTGTTCACTATGCC-3'; VTN Reverse: 5'-GTGTCTGCTCAGGATTCCCTT-3'; VGF Forward: 5'-GGAACTGCGAGATTTCAGTCC-3'; VGF Reverse: 5'-GTGCGGGTTTCCGTCTCTG-3'; MFI2 Forward: 5'-ACCTCCTATTACGCCGTGG-3'; MFI2 Reverse: 5'-AGGGACTCAGAGTAACTGGTC-3'; SAA1 Forward: 5'-CATGCTCGGGGGAACTAT-3'; SAA1 Reverse: 5'-TACCCATTGTGTACCCTCTCC-3'.

### Western blotting (WB)

In brief, the proteins of tissues were extracted with the radio-immunoprecipitation assay (RIPA) lysis buffer (Beyotime, China) containing 1mM Phenylmethylsulfonyl fluoride (PMSF, Beyotime, China). The concentration of protein was detected using the BCA assay kit (MCE, China). 50ug of protein was subjected to 10% SDS-PAGE and transferred to PVDF membranes (Millipore, USA). Then, the membranes were blocked in 5% nonfat dried skimmed milk for 1.5h at room temperature. After that, the PVDF membranes were incubated with primary antibodies containing VGF (1:1000, Abcam, England, ab74140) and GAPDH (1:50000, Abclonal, China, AC002) overnight at 4 °C. Finally, the membranes were incubated with corresponding secondary antibodies (1:3000, Proteintech, China, SA00001-1 and SA00001-2) for 1.5h at room temperature and visualized with the ChemiDoc-XRS+ system (Bio-Rad, USA).

### Immunohistochemistry (IHC) assay

IHC was performed as previously described 39. Firstly, ccRCC samples were fixed in formalin, dehydrated, and embedded in paraffin. Secondly, sample sections were incubated with corresponding primary antibodies overnight at 4 °C. Thirdly, sample sections were washed with PBS three times. Finally, sample sections were incubated with corresponding secondary antibodies at room temperature for 2h. Moreover, ImageJ software was used to calculate the mean OD value of cells with positive staining.

The primary antibodies and secondary antibodies were as following: MMP9 (1:100, Abclonal, China, A0289); IGFBP1 (1:150, Abclonal, China, A11109); CD14 (1:200, Proteintech, China, 60253-1-lg); secondary antibodies (1:3000, Proteintech, China, SA00001-1 and SA00001-2).

### Statistical analysis

All data were presented as mean ± SD with three independent experiments. The student's t test was used for two group data. Analysis of variance (ANOVA) was used to analyze multiple groups of data and least - significant difference (LSD) was used to analyze the difference between groups. All statistical analyses were performed using GraphPad Prism software (GraphPad Software, Inc., La Jolla, CA, USA). P < 0.05 was considered to be statistically significant.

## Results

### Distribution of TIICs in ccRCC and prognostic role of TIICs

**Figure 2:**
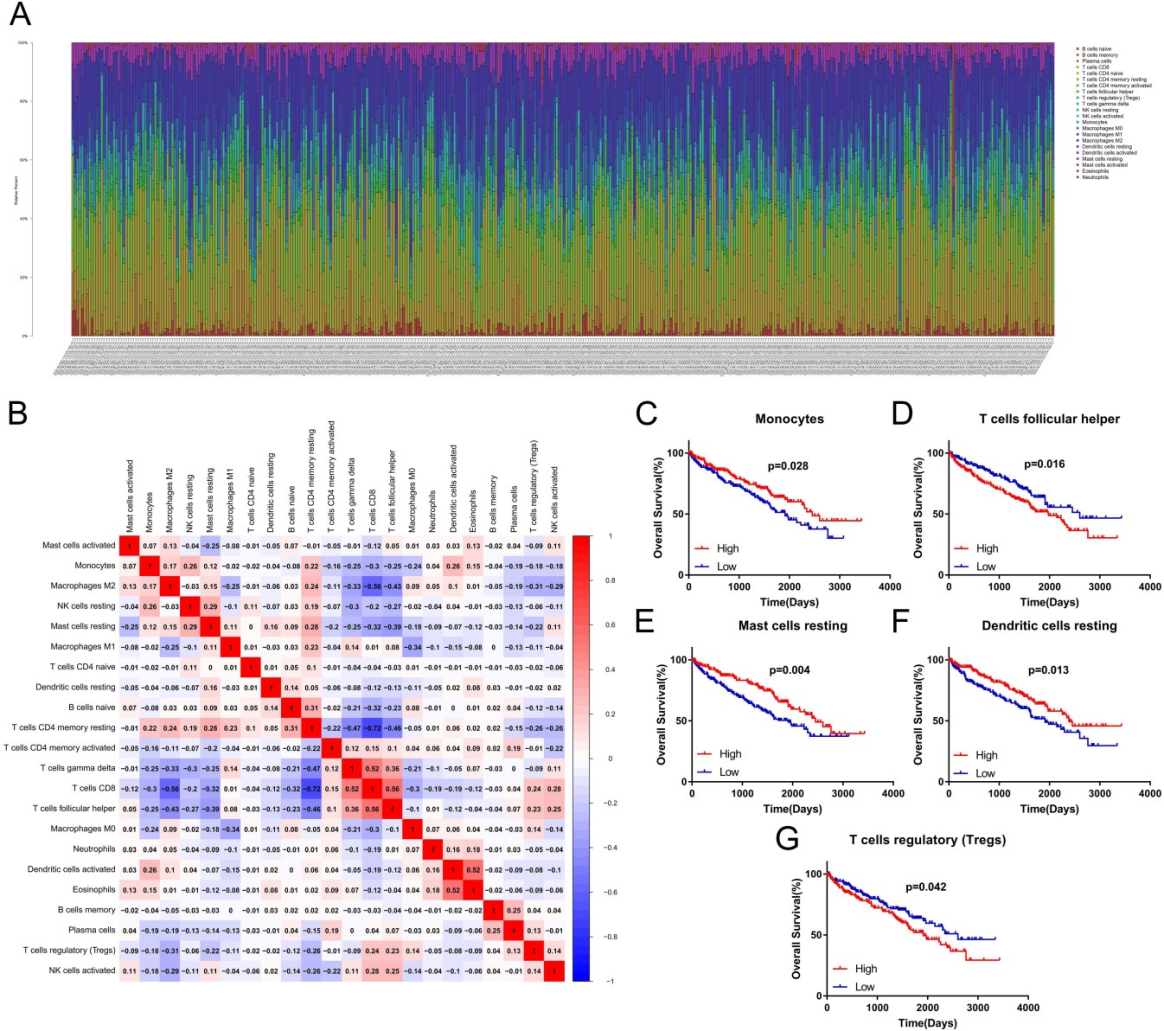
** Distribution and prognostic role of TIICs in ccRCC.** (A) Distribution of 22 TIICs in the TCGA KIRC dataset. (B) The heat map of correlation among 22 TIICs. (C-G) The prognostic value of TIICs. TIICs: tumor-infiltrating immune cells; ccRCC: clear cell renal cell carcinoma; TCGA: The Cancer Genome Atlas; KIRC: Kidney Renal Clear Cell Carcinoma.

CIBERSORT was performed to identify the landscape of 22 TIICs in ccRCC. As shown in Figure 2A, tumors contained abundant fractions of CD8+ T cells (21.5%), M2 macrophages (20.3%), resting memory CD4+ T cells (16.1%), M1 macrophages (8.4%) and monocytes (4.9%), whereas the fractions of naive CD4+ T cells (0.00%), memory B cells (0.02%), eosinophils (0.05%) and activated dendritic cells (0.13%) were low. The correlation heat map showed that the proportions of different TIICs subtypes were not strongly correlated (Figure 2B). Moreover, survival analysis displayed that high proportion of monocytes (p=0.028, HR=0.68), resting mast cells (resting MCs) (p=0.004, HR=0.61) and resting dendritic cells (resting DCs) (p=0.013, HR=0.65) predicted better OS (Figure 2C, E, F). However, patients with high proportion of follicular helper T (Tfh) cells (p=0.016, HR=1.51) or regulatory T cells (Tregs) (p=0.042, HR=1.42) had shorter OS time (Figure 2D, G).

### Construction of Cox proportional hazard regression model

**Figure 3:**
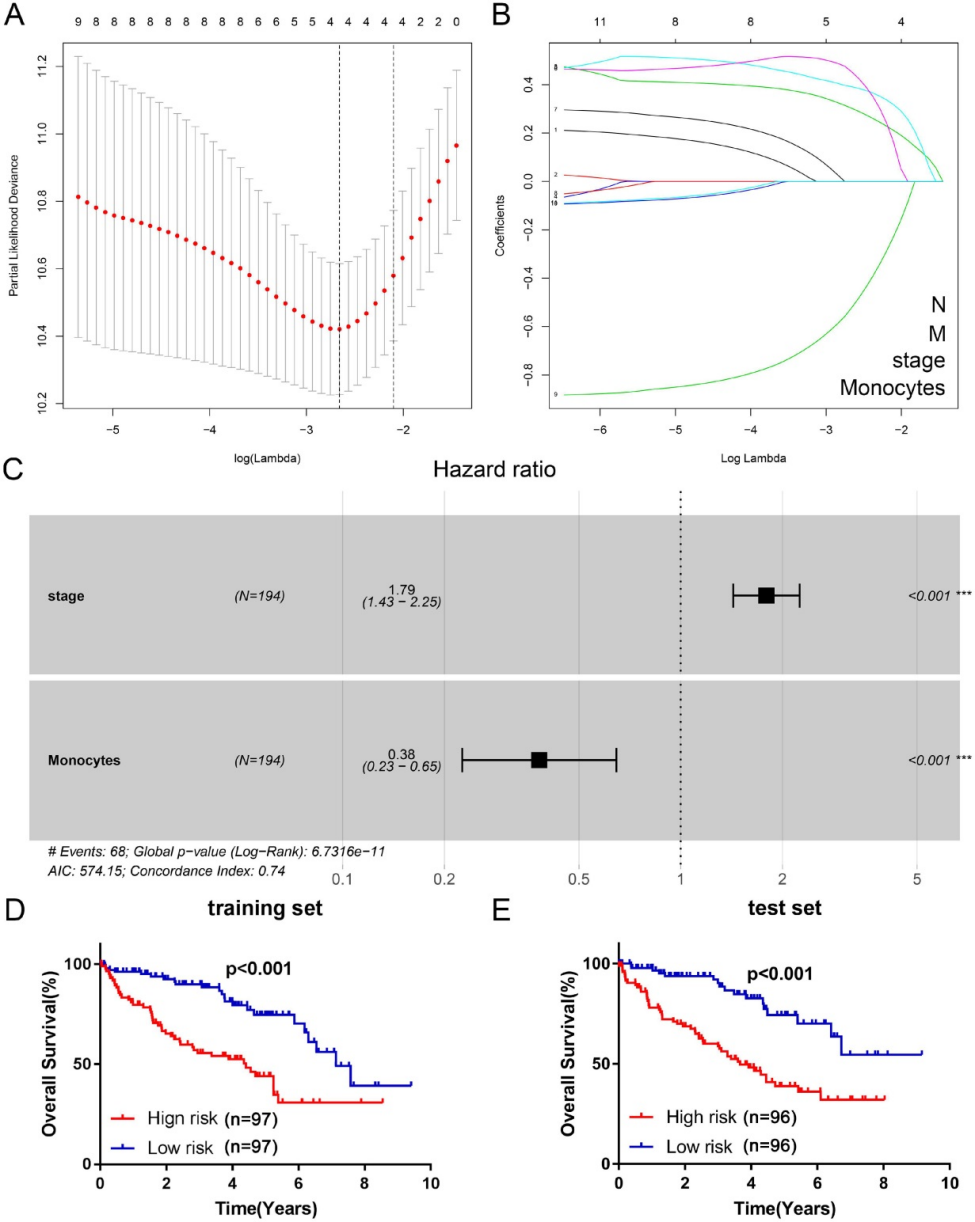
** Construction of the Cox regression proportional hazard model.** (A) Partial likelihood deviance for the LASSO coefficient profiles. (B) LASSO coefficient profiles of the clinical traits and TIICs. (C) Multivariate cox regression analysis of four variates (N, M, stage, and monocytes). Survival analysis was performed to verify the prognostic role of risk index for training set (D) and test set (E). LASSO: least absolute shrinkage and selection operator; TIICs: tumor-infiltrating immune cells.

Firstly, we used univariate cox proportional hazard regression model to preliminarily screen important clinicopathological characteristics and TIICs which are related to OS in training set. Univariate analysis showed that age (p=0.079, HR=1.6, CI: 0.95-2.70), grade (p=0.001, HR=1.79, CI: 1.27-2.52), TNM stage (p=0.000, HR=1.83, CI: 1.47-2.27), T stage (p=0.000, HR=1.82, CI: 1.40-2.36), N stage (p=0.000, HR=5.19, CI: 2.46-10.98), M stage (p=0.000, HR=4.21, CI: 2.53-7.00), plasma cells (p=0.074, HR=1.55, CI: 0.96-2.52), regulatory T cells (p=0.025, HR=1.76, CI: 1.07-2.88), monocytes (p=0.000, HR=0.39, CI: 0.23-0.64), resting dendritic cells (p=0.047, HR=0.61, CI: 0.37-0.99) and resting mast cells (p=0.063, HR=0.63, CI: 0.39-1.03) were significantly related to prognosis (Table 1). Then, based on the LASSO cox regression model, four indexes (TNM stage, N stage, M stage and monocytes) with nonzero coefficient were selected as candidate prognostic indexes (Figure 3A, B). Finally, as shown in Figure 3C, TNM stage (coefficient=0.58, HR=1.79, CI: 1.43-2.25, p<0.001) and monocytes (coefficient=-0.96, HR=0.38, CI: 0.23-0.65, p<0.001) were selected to construct risk index and prognostic signature via multivariate cox regression analysis. The risk index = 0.58*TNM stage - 0.96*Monocytes. Then, we divided ccRCC patients into two clusters (high risk/TNM^high^Monocytes^low^ cluster and low risk/TNM^low^Monocytes^high^ cluster) in both training set and test set according to the risk score. Survival analysis indicated that patients with high risk index had worse prognosis (Figure 3D, E) both in training set (HR=2.96, CI: 1.82-4.83, p<0.001) and test set (HR=3.03, CI: 1.88-4.88, p<0.001).

### Identification of DEGs between high risk/TNM^high^Monocytes^low^ cluster and low risk/TNM^low^Monocytes^high^ cluster

**Figure 4:**
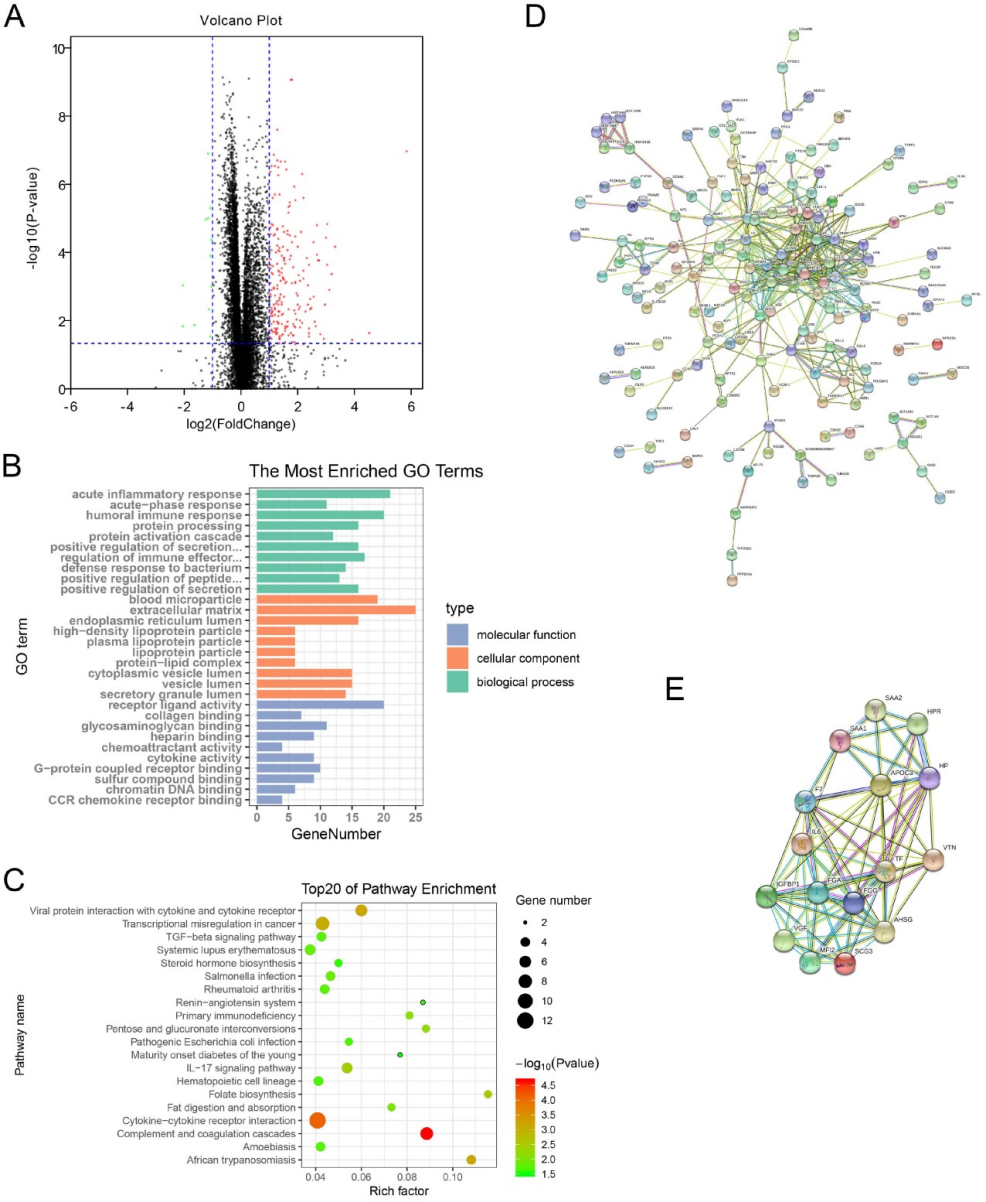
** Functional annotation and PPI network analysis of DEGs.** (A) Identification of DEGs between high-risk group and low-risk group. Red plots represent up-regulated genes, green plots represent down-regulated genes, and black plots represent unchanged genes. (B) GO annotation of DEGs. (C) KEGG pathway enrichment analysis of DEGs. (D) PPI network analysis of DEGs. (E) Identification of key modules via MCODE plug. PPI: protein-protein interaction; DEGs: differentially expressed genes; GO: Gene Ontology; KEGG: Kyoto Encyclopedia of Genes and Genomes; MCODE: Molecular Complex Detection.

A total of 216 genes were identified as DEGs with |log FC| > 1.0 and P-value < 0.05. There were 204 up-regulated genes and 12 down-regulated genes in the high-risk cluster (Figure 4A).

### Function enrichment analysis of DEGs

GO enrichment analysis indicated that the DEGs were mostly enriched in acute inflammatory response, humoral immune response, and regulation of immune effector process in the biological process (BP) group. Cellular component (CC) analysis showed that the DEGs were significantly enriched in blood microparticle, extracellular matrix, and endoplasmic reticulum lumen. Furthermore, molecular function (MF) results displayed that the DEGs were mainly related to receptor ligand activity, cytokine activity, and CCR chemokine receptor binding (Figure 4B). Moreover, KEGG pathway enrichment analysis also showed that the DEGs were obviously enriched in cytokine-cytokine receptor interaction, IL-17 signaling pathway, and TGF-beta signaling pathway. It was worth mentioning that the DEGs were enriched in salmonella infection and pathogenic Escherichia coli infection pathways (Figure 4C).

### Construction of PPI network and identification of hub genes

All DEGs were uploaded to STRING online database for constructing the PPI network. There were 157 nodes and 436 edges in the PPI network (Figure 4D). As shown in Figure 4E, the most important module contained 16 nodes (MFI2, SAA2, SCG3, AHSG, VTN, HP, IGFBP1, TF, IL6, FGG, HPR, FGA, VGF, F2, APOC3, and SAA1) and 76 edges. According to degree > 10, a total of 24 genes were identified as hub genes (Table 2).

### Survival analysis of hub genes

**Figure 5:**
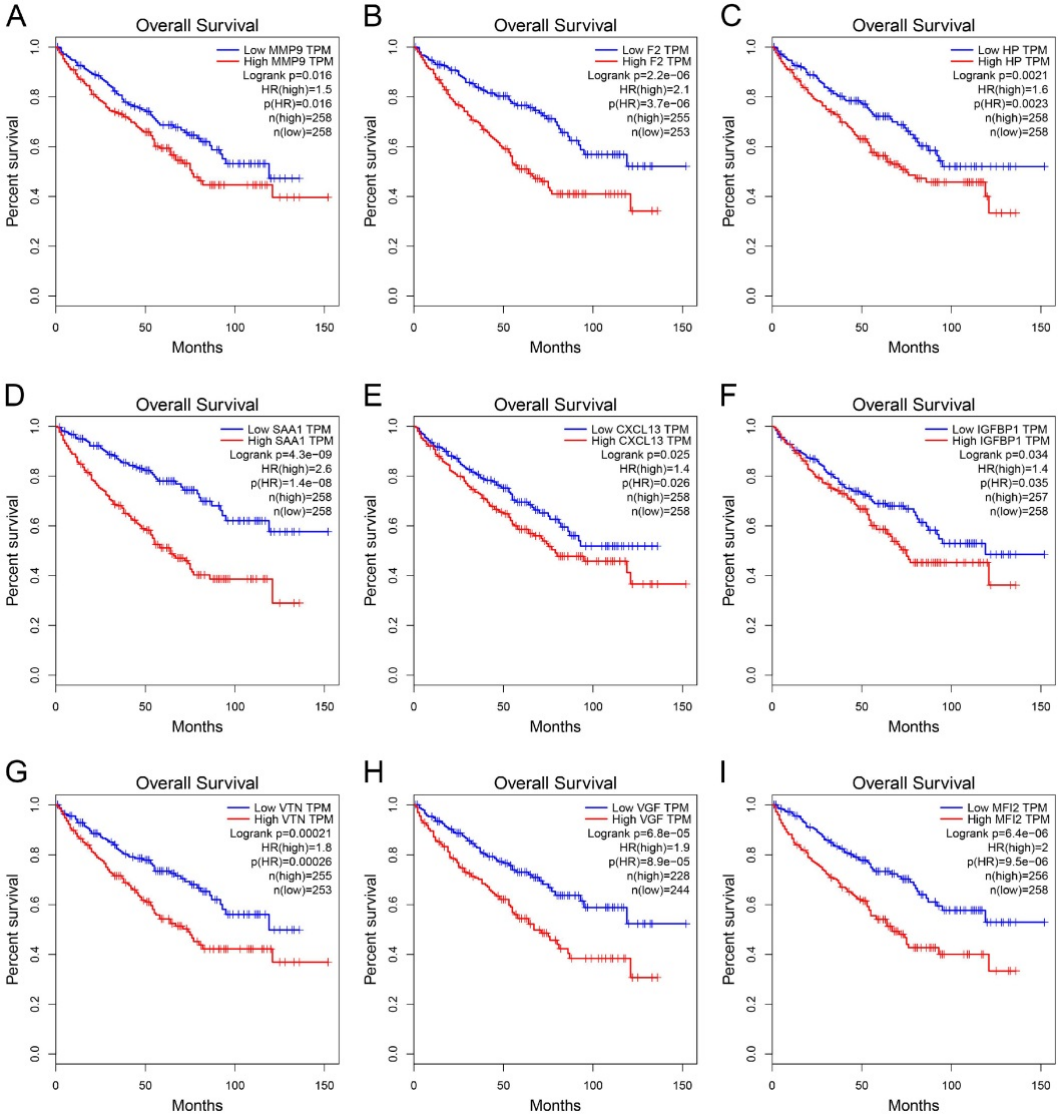
** Survival analysis of hub genes for OS.** (A) MMP9, Logrank p=0.016, HR=1.5. (B) F2, Logrank p=2.2e-06, HR=2.1. (C) HP, Logrank p=0.0021, HR=1.6. (D) SAA1, Logrank p=4.3e-09, HR=2.6. (E) CXCL13, Logrank p=0.025, HR=1.4. (F) IGFBP1, Logrank p=0.034, HR=1.4. (G) VTN, Logrank p=0.00021, HR=1.8. (H) VGF, Logrank p=6.8e-05, HR=1.9. (I) MFI2, Logrank p=6.4e-05, HR=2.0. OS: overall survival; HR: hazard ratio.

**Figure 6:**
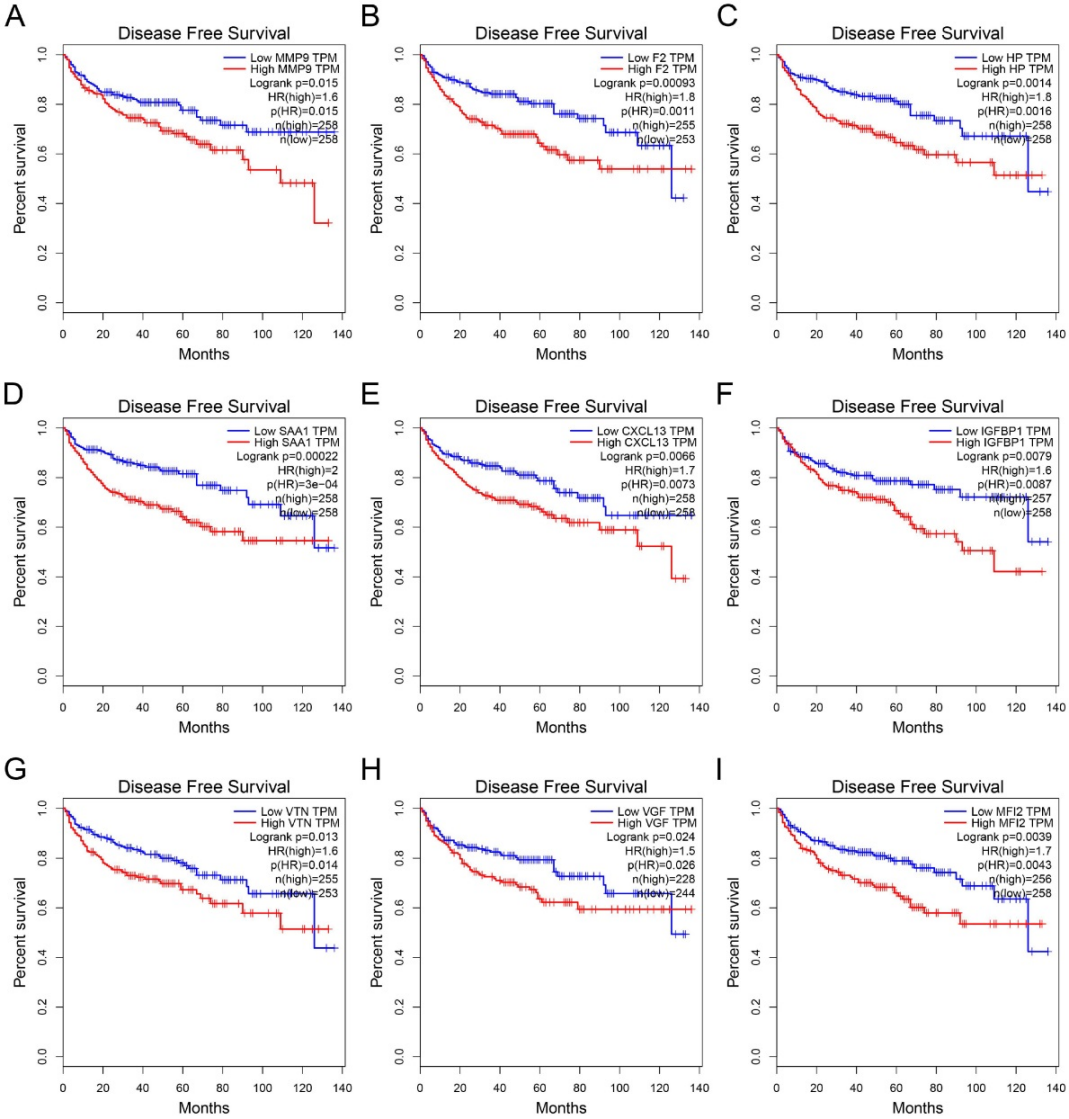
** Survival analysis of hub genes for DFS.** (A) MMP9, Logrank p=0.015, HR=1.6. (B) F2, Logrank p=0.00093, HR=1.8. (C) HP, Logrank p=0.0014, HR=1.8. (D) SAA1, Logrank p=0.00022, HR=2.0. (E) CXCL13, Logrank p=0.0066, HR=1.7. (F) IGFBP1, Logrank p=0.0079, HR=1.6. (G) VTN, Logrank p=0.013, HR=1.6. (H) VGF, Logrank p=0.024, HR=1.5. (I) MFI2, Logrank p=0.0039, HR=1.7. DFS: disease-free survival; HR: hazard ratio.

GEPIA was used to perform survival analysis (OS and DFS) of 24 hub genes. As shown in Figure 5 and Figure 6, high expression of MMP9, F2, HP, SAA1, CXCL13, IGFBP1, VTN, VGF, and MFI2 predicted poor OS and DFS.

### Validation of hub genes in ccRCC

**Figure 7:**
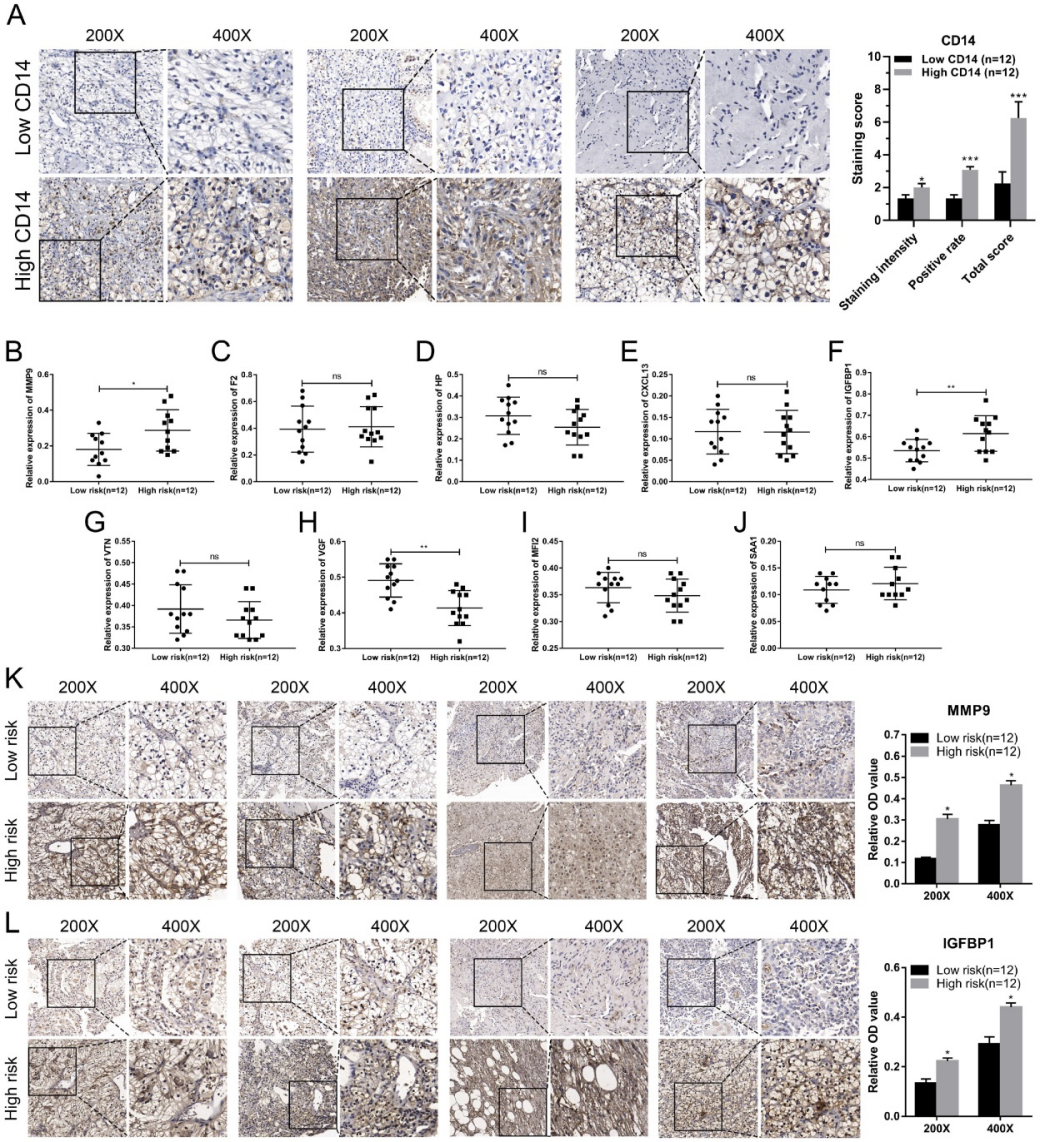
** Validation of hub genes in the high-risk group and low-risk group.** (A) ccRCC samples were divided into high monocytes group and low monocytes group based on the expression of CD14. Validation of the mRNA expression of MMP9 (B), F2 (C), HP (D), CXCL13 (E), IGFBP1 (F), VTN (G), VGF (H), MFI2 (I), and SAA1(J) between high-risk group and low-risk group. IHC assay was used to verify the protein expression of MMP9 (K) and IGFBP1 (L) in ccRCC. ccRCC: clear cell renal cell carcinoma. ccRCC: clear cell renal cell carcinoma.

**Figure 8:**
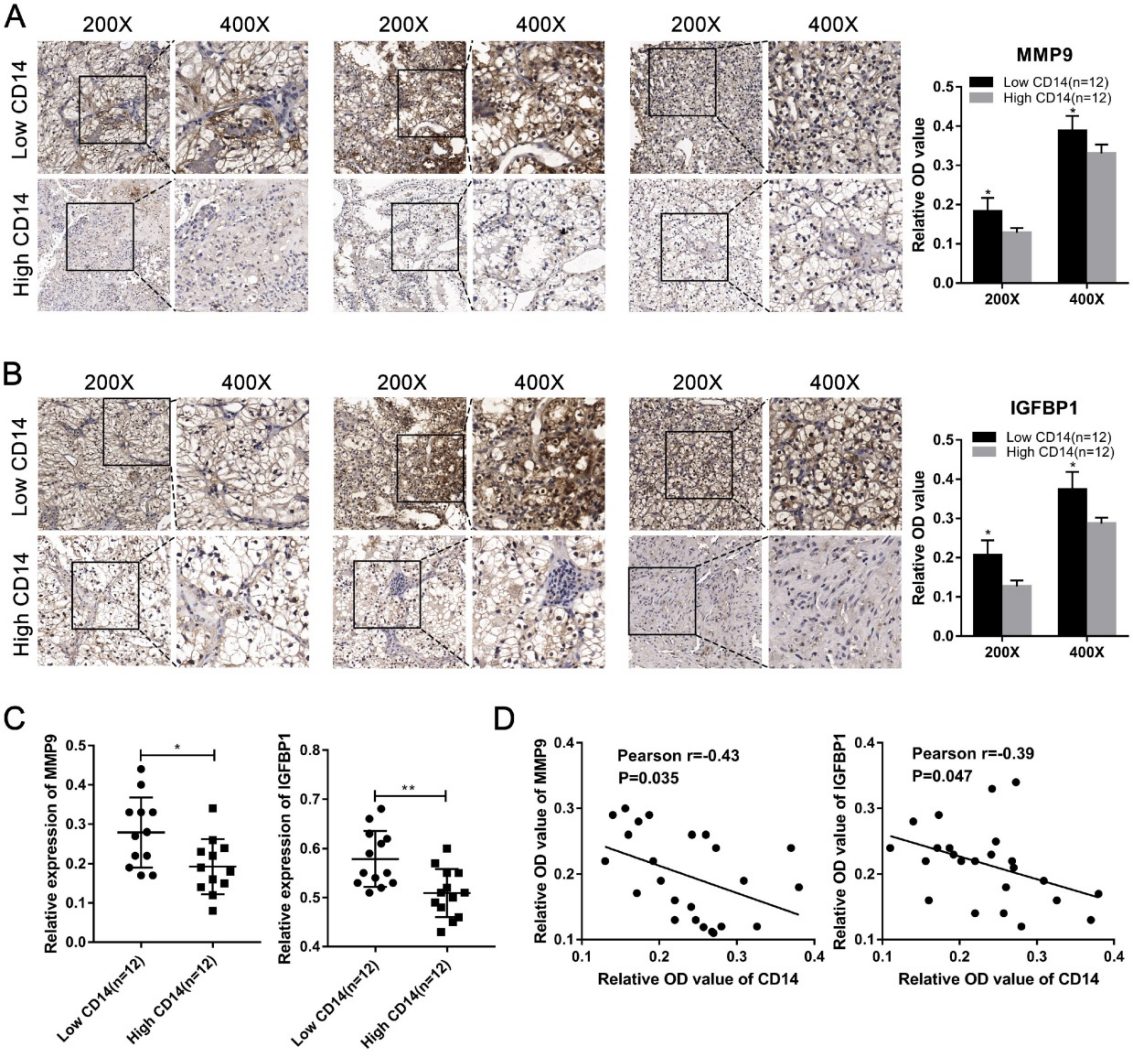
** MMP9 and IGFBP1 were up-regulated in the monocytes^low^ group and negative correlated to CD14.** (A-C) Both protein expression and mRNA levels of MMP9/IGFBP1 were elevated in the monocytes^low^ group. (D) The protein expression levels of MMP9 (Pearson r=-0.0.43, P=0.035) and IGFBP1 (Pearson r=-0.39, P=0.047) were negative correlated to CD14.

We utilized the IHC results of monocytes' signature (CD14) to identify the relative abundance of monocytes in ccRCC tissues (Figure 7A). Combined with the TNM stage, all ccRCC tissues were divided into high-risk group and low-risk group according to the risk index formula (Table 3). Then, qRT-PCR assay was used to validate the mRNA expression level of hub genes between high-risk group and low-risk group. As shown in Figure 7B-J, we found that the mRNA expressions of MMP9 and IGFBP1 were elevated in the high-risk group, and VGF was down-regulated in the high-risk group. However, differential analysis showed that VGF was up-regulated in the high-risk group, which was inconsistent with qRT-PCR results. Further WB assay showed that there was no significant difference between high risk group and low risk group (Figure S1). Therefore, we selected MMP9 and IGFBP1 for subsequent analysis. IHC results further showed that the protein expression level of MMP9 and IGFBP1 were up-regulated in the high-risk group (Figure 7K-L). Furthermore, the ccRCC samples were also divided into monocytes^high^ group and monocytes^low^ group according to the IHC results of CD14. We found that the expression level of MMP9 and IGFBP1 were elevated in the monocytes^low^ group (Figure 8A-C). Further correlation analysis showed that both MMP9 (Pearson r=-0.0.43, P=0.035) and IGFBP1 (Pearson r=-0.39, P=0.047) were negatively correlated to CD14 (Figure 8D). These findings indicated that MMP9 and IGFBP1 were involved in tumor immune and progression.

### GSEA of MMP9 and IGFBP1

**Figure 9:**
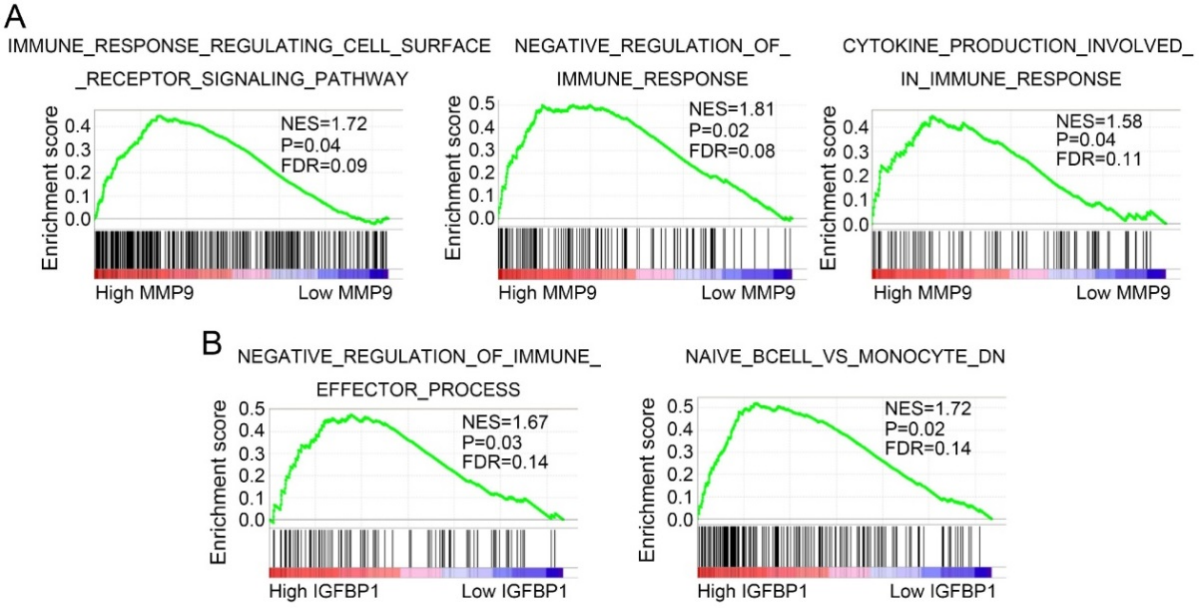
** MMP9 and IGFBP1 regulate immune related pathways.** GSEA was performed to discover the potential mechanisms of MMP9 (A) and IGFBP1 (B) in ccRCC. GSEA: gene set enrichment analysis; ccRCC: clear cell renal cell carcinoma.

GSEA was performed to discover potential molecular mechanisms of MMP9 and IGFBP1 in tumor immune and progression. As shown in Figure 9A, MMP9 was mainly enriched in immune response regulating cell surface receptor signaling pathway, negative regulation of immune response pathway, and cytokine production involved in immune response pathway. In addition, IGFBP1 was significantly associated with negative regulation of immune effector process pathway and naive B cell vs monocyte pathway (Figure 9B).

## Discussion

Nowadays, immunotherapy is used to treat advanced ccRCC or metastatic ccRCC, which prolong the overall survival time of patients. Many studies have shown that immunotherapy can activate the immune system and inhibit tumor progression 40-42. Unfortunately, there are still many patients whose state was not improved due to drug resistance and influence of tumor microenvironment (TME). At present, we recognize that tumor is not simply a cell mass composed of malignant cells but is actually composed of many non-malignant cells 43,44. Tumor-infiltrating immune cell (TIIC) is one of the most important non-malignant cells, which involved in regulating tumor immune and immune escape. Several reports also revealed that TIICs were significantly correlated to the expression of immune checkpoints 45,46. Therefore, further study on the role of TIICs may improve the efficacy of immunotherapy and find new therapeutic targets.

In our study, the abundances of 22 TIICs were identified based on the gene sequencing data. Then, monocytes and the TNM stage were identified as independent prognostic factors and constructed the prognostic signature via cox regression analysis in ccRCC. Further studies uncovered that MMP9 and IGFBP1 were associated with tumor immune and progression.

Monocytes are mononuclear phagocytes that play an important role in the inflammatory response, immune response, and maintaining tissue homeostasis 47. Recently, increasing studies indicated that tumor-infiltrating monocytes were significantly correlated to tumor progression and displayed diverse functions at different stages of tumor. Liu *et al* reported that CCL15 could recruit CCR1^+^CD14^+^ monocytes to promote tumor immune escape and progression 48. Similarly, Qian *et al* revealed that CCL2 facilitated tumor metastasis through recruiting Gr1-positive inflammatory monocytes in breast cancer 49. It has been reported that tumor-infiltrating monocytes promoted invasion and migration of tumor cell via up-regulating S100A8 and S100A9 expression 50. Monocytes also could differentiate into tumor-associated macrophages (TAM) to drive tumor growth and metastasis 51. In addition, monocytes also acted as a tumor suppressor through recruiting NK cells 52,53, inhibiting Tregs function 54, and regulating cytotoxicity 55,56. In the present study, we also found that monocytes served as an independent prognostic factor and ccRCC patients with high abundance of monocytes predicted better OS. These findings suggested that tumor-infiltrating monocytes may suppress ccRCC progression. Then, differential analysis and PPI network analysis indicated that MMP9 and IGFBP1 may act as mediators of monocytes in ccRCC.

MMP9 (matrix metallopeptidase 9), a member of matrix metallopeptidase superfamily, is mainly involved in local proteolysis of the extracellular matrix and leukocyte migration 57,58. Numerous studies verified that MMP9 promoted tumor growth and metastasis in various cancers 59-61. Furthermore, previous studies reported that MMP9 could regulate the biological functions of monocytes and their differential cells 62-65. In this study, we also found that MMP9 was elevated in the TNM^high^Monocytes^low^ group of ccRCC. Meanwhile, GSEA showed that high MMP9 group was significantly enriched in immune response pathway, cell surface receptor signaling pathway, and cytokine production pathway. These findings suggested that MMP9 may act as a regulator of cytokines and membrane receptors to mediate immune response in ccRCC.

IGFBP1 (insulin like growth factor binding protein 1) is a member of IGFBPs superfamily, which mainly regulates the bioavailability of IGF-1 and synthesizes in the liver and kidney 66,67. It has been reported that IGFBP1 plays a dual role in both tumor growth and metastasis 68-70. Moreover, previous studies revealed that IGFBP1 was involved in regulating cell migration 71. Brandt *et al* uncovered that IGFBP1 and its fragments promoted human dermal fibroblasts migration 72. Dorniak *et al* demonstrated that prostaglandins stimulated trophectoderm cell migration and attachment via regulating IGFBP1 expression 73. Similarly, Irving et al also proved that IGFBP1 regulated the migration function of first trimester invasive trophoblasts by interacting with the RGD binding site of the α-5-β-1 integrin 74. These studies suggested that IGFBP1 was significantly associated with tumor progression and cell migration. In the present study, IGFBP1 was identified as a regulator for tumor progression and immune in ccRCC. Furthermore, GSEA uncovered that IGFBP1 was mainly involved in negative regulating of immune effector process pathway and naïve B cell vs monocyte pathway. These results indicated that IGFBP1 might regulate tumor progression and immune via mediating the biological functions of monocytes.

In conclusion, monocyte was identified as an independent protective factor in ccRCC. Further bioinformatics analysis revealed that MMP9 and IGFBP1 might act as regulators for monocytes migration and tumor immune. Moreover, GSEA indicated that MMP9 regulated immune response by mediating cell surface receptor and cytokine pathways and IGFBP1 was mainly involved in the regulation of monocytes. These findings suggested that MMP9 and IGFBP1 could become potential biomarkers and therapeutic targets for immunotherapy in ccRCC.

## Supplementary Material

Supplementary figure S1.

## Figures and Tables

**Table 1:** Univariate cox regression analysis of TIICs

Variate	HR	95%CI Low	95%CI High	P-value
Age	1.60	0.95	2.70	0.079
Gender	0.85	0.52	1.39	0.517
Grade	1.79	1.27	2.52	0.001
Stage	1.83	1.47	2.27	0.000
T	1.82	1.40	2.36	0.000
M	4.21	2.53	7.00	0.000
N	5.19	2.46	10.98	0.000
B cells naive	0.99	0.61	1.59	0.951
Plasma cells	1.55	0.96	2.52	0.074
T cells CD8	0.75	0.46	1.21	0.234
T cells CD4 memory resting	0.87	0.54	1.40	0.556
T cells follicular helper	1.19	0.74	1.94	0.473
T cells regulatory (Tregs)	1.76	1.07	2.88	0.025
T cells gamma delta	0.71	0.44	1.15	0.163
NK cells activated	1.03	0.64	1.65	0.916
Monocytes	0.39	0.23	0.64	0.000
Macrophages M0	1.27	0.79	2.06	0.329
Macrophages M1	0.73	0.45	1.18	0.201
Macrophages M2	0.84	0.52	1.36	0.474
Dendritic cells resting	0.61	0.37	0.99	0.047
Mast cells resting	0.63	0.39	1.03	0.063

TIICs: tumor-infiltrating immune cells

**Table 2:** PPI network of hub genes

Gene	Degree	Betweenness Centrality	Closeness Centrality	Clustering Coefficient	Neighborhood Connectivity	Stress	Differential Analysis	
							log FC	P-value
IL6	42	0.42	0.48	0.16	10.67	23056	1.08	0.000
MMP9	27	0.12	0.41	0.21	11.85	8260	1.79	0.000
F2	25	0.08	0.41	0.31	14.52	5774	1.97	0.000
HP	24	0.03	0.38	0.43	15.38	3242	2.65	0.000
APOA1	23	0.03	0.40	0.48	15.26	3186	1.40	0.026
ORM1	22	0.04	0.36	0.39	13.73	4358	2.89	0.032
SAA1	22	0.06	0.40	0.35	14.45	4770	2.68	0.00
FGG	20	0.02	0.39	0.53	16.00	2544	1.42	0.012
AHSG	20	0.02	0.40	0.54	17.10	2894	2.37	0.023
APOC3	18	0.04	0.38	0.56	17.17	2526	2.48	0.004
FGA	18	0.01	0.38	0.59	17.11	1978	1.87	0.005
TF	17	0.01	0.39	0.61	17.41	1618	1.77	0.043
ORM2	15	0.00	0.33	0.63	16.33	364	1.65	0.026
CD19	14	0.08	0.37	0.32	10.00	5626	1.27	0.001
CXCL13	14	0.03	0.37	0.46	13.64	2362	1.78	0.000
IGFBP1	14	0.01	0.38	0.58	17.93	1150	1.42	0.000
CCL19	13	0.01	0.37	0.54	14.69	1600	1.34	0.000
CCL20	13	0.01	0.36	0.51	14.00	1264	1.02	0.030
VTN	13	0.02	0.36	0.60	19.15	1582	2.40	0.017
SCG3	11	0.04	0.38	0.69	17.45	4654	1.90	0.015
VGF	11	0.05	0.38	0.67	17.00	5026	1.93	0.001
IFNG	11	0.03	0.37	0.38	15.18	2332	1.20	0.000
MFI2	11	0.02	0.36	0.67	16.45	876	1.85	0.003
SAA4	11	0.00	0.32	0.84	18.09	78	2.60	0.014

PPI: protein - protein interaction; FC: fold change

**Table 3:** The risk group of ccRCC samples

Patient ID	TNM stage	Monocyte/dummy variable^Ѱ^	Risk score/Risk group^Ѧ^
Case-1	2	0.253/2	-0.76/Low
Case-2	3	0.201/2	-0.18/Low
Case-3	1	0.248/2	-1.34/Low
Case-4	2	0.210/2	-0.76/Low
Case-5	4	0.164/1	1.36/High
Case-6	2	0.189/1	0.20/High
Case-7	3	0.176/1	0.78/High
Case-8	4	0.168/1	1.36/High
Case-9	2	0.265/2	-0.76/Low
Case-10	1	0.241/2	-1.34/Low
Case-11	1	0.232/2	-1.34/Low
Case-12	2	0.201/2	-0.76/Low
Case-13	4	0.173/1	1.36/High
Case-14	2	0.198/1	0.20/High
Case-15	3	0.186/1	0.78/High
Case-16	3	0.157/1	0.78/High
Case-17	1	0.221/2	-1.34/Low
Case-18	2	0.158/1	0.20/High
Case-19	4	0.162/1	1.36/High
Case-20	2	0.206/2	-0.76/Low
Case-21	3	0.190/1	0.78/High
Case-22	1	0.259/2	-1.34/Low
Case-23	2	0.272/2	-0.76/Low
Case-24	4	0.191/1	1.36/High

dummy variable^Ѱ^: 1 represents the relative abundance of monocyte less than the median; 2 represents the relative abundance of monocyte more than the median. Risk group^Ѧ^: High represents the risk score of patient less than the median; Low represents the risk score of patient more than the median.

## Abbreviations

ccRCC: clear cell renal cell carcinoma
TIICs: tumor-infiltrating immune cells
PPI: protein-protein interaction
RCC: renal cell carcinoma
mTOR: mammalian target of rapamycin
TME: tumor microenvironment
ECM: extracellular matrix
DCs: dendritic cells
NK: natural killer
TCGA KIRC: The Cancer Genome Atlas Kidney Renal Cell Carcinoma
DEGs: differentially expressed genes
OS: overall survival
LASSO: least absolute shrinkage and selection operator
GO: Gene Ontology
KEGG: Kyoto Encyclopedia of Genes and Genomes
MCODE: Molecular Complex Detection
GSEA: Gene set enrichment analysis
qRT-PCR: quantitative realtime PCR
IHC: Immunohistochemistry
ANOVA: Analysis of variance
LSD: least - significant difference
MCs: mast cells
Tfh: follicular helper T
Tregs: regulatory T cells
FC: fold change
BP: biological process
CC: cellular component
MF: molecular function
TAM: tumor-associated macrophages
