# Supplementary material for: MMP9 and IGFBP1 Regulate Tumor Immune and Drive Tumor Progression in Clear Cell Renal Cell Carcinoma
Source: J Cancer. 2021 Feb 22;12(8):2243–57. doi: 10.7150/jca.48664 (PMC7974879; doi:10.7150/jca.48664)
Supplement: Supplementary file 1 — Supplementary figure S1. [file jcav12p2243s1.pdf]

Supplementary material

Supplementary Figure

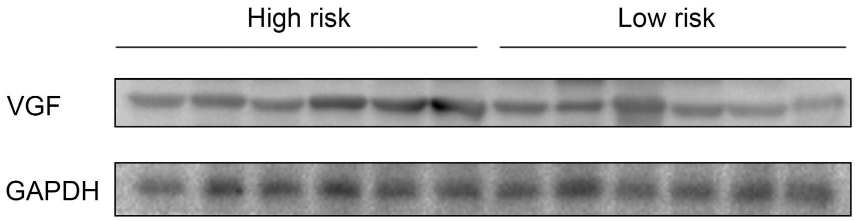

FigureS1. Validation of the protein expression of VGF between high risk group and low risk group. There is no significant difference between the two groups.
